# Assessing a cut-off point for the diagnosis of abnormal uterine bleeding using the Menstrual Bleeding Questionnaire (MBQ): a validation and cultural translation study with Brazilian women

**DOI:** 10.1590/1516-3180.2022.0539.R2.100423

**Published:** 2023-07-07

**Authors:** Gabriela Pravatta Rezende, Luiz Gustavo Oliveira Brito, Daniela Angerame Yela Gomes, Leticia Mansano de Souza, Sergio Polo, Cristina Laguna Benetti-Pinto

**Affiliations:** I MD, MSc. Attending Physician, Department of Tocogynecology, School of Medical Sciences, Universidade Estadual de Campinas (UNICAMP), Campinas (SP), Brazil.; II MD, PhD. Associate Professor, Department of Tocogynecology, School of Medical Sciences, Universidade Estadual de Campinas (UNICAMP), Campinas (SP), Brazil.; III MD, PhD. Associate Professor, Department of Tocogynecology, School of Medical Sciences, Universidade Estadual de Campinas (UNICAMP), Campinas (SP), Brazil.; IV Medical Student, School of Medical Sciences, Universidade Estadual de Campinas (UNICAMP), Campinas (SP), Brazil.; V Medical Student, School of Medical Sciences, Universidade Estadual de Campinas (UNICAMP), Campinas (SP), Brazil.; VI MD, PhD. Associate Professor, Department of Tocogynecology, School of Medical Sciences, Universidade Estadual de Campinas (UNICAMP), Campinas (SP), Brazil.

**Keywords:** Metrorrhagia, Translations, Validation study [publication type], Abnormal uterine bleeding, Menstrual bleeding questionnaire, Prospective cohort study

## Abstract

**BACKGROUND:**

Abnormal uterine bleeding (AUB) is a common condition, and the Menstrual Bleeding Questionnaire (MBQ) is used for its assessment.

**OBJECTIVES:**

To translate, assess the cut-off point for diagnosis, and explore psychometric properties of the MBQ for use in Brazilian Portuguese.

**DESIGN AND SETTING:**

Prospective cohort study including 200 women (100 with and 100 without AUB) at a tertiary referral center.

**METHODS:**

MBQ translation involved a pilot-testing phase, instrument adjustment, data collection, and back-translation. Cut-off point was obtained using receiver operating curve analysis. Menstrual patterns, impact on quality of life due to AUB, internal consistency, test-retest, responsiveness, and discriminant validity were assessed. For construct validity, the Pictorial Blood Assessment Chart (PBAC) and World Health Organization Quality of Life – abbreviated version (WHOQOL-BREF) were applied.

**RESULTS:**

Women with AUB were older, had higher body mass indices, and had a worse quality of life during menstruation. Regarding the MBQ's psychometric variables, Cronbach's alpha coefficient was > 0.70 in all analyses, high intraclass correlation coefficient was found in both groups; no ceiling and floor effects were observed, and construct validity was demonstrated (correlation between MBQ score, PBAC score, and clinical menstrual cycle data). No difference between MBQ and PBAC scores were perceived after the test-retest. Significant differences were found between MBQ and PBAC scores before and after treatment. An MBQ score ≥ 24 was associated with a high probability of AUB; accuracy of 98%.

**CONCLUSION:**

The MBQ is a reliable questionnaire for Brazilian women. The cut-off ≥ 24 shows high accuracy to discriminate AUB.

## INTRODUCTION

Abnormal uterine bleeding (AUB) is defined by menstrual cycle changes, in regularity, volume, frequency, or duration in non-pregnant women.^1,2^ Menstrual disorders represent the leading cause of seeking gynecological care; additionally, it is estimated that up to 40% of women are affected by this condition during their lifetime.^3–6^ AUB may have a negative impact on economic productivity, relationships, quality of life (QOL), and increasing costs for health services.^7–9^

The approach for AUB is based on a clinical assessment. Studies that evaluated the assistance provided to these women showed that the classic clinical approach does not always include parameters considered important by women with this condition.^10^ For example, difficulty in predicting when bleeding will occur and the chance of blood spilling onto clothing can cause discomfort. Also, excessive blood loss can lead to embarrassing experiences and symptoms that are not always considered in a traditional medical evaluation.^10^ Few publications have reported experiences and feelings of women during the menstrual and intermenstrual period (“patient self-reported outcomes”), with reviews confirming that the classic model of medical care may be unable to understand the entire context experienced by patients. Recent research recognizes the importance of evaluating not only the response to the treatments related to blood loss reduction but also the experience of women. The Institute National Health and Care Unit of the United Kingdom recommendations reinforce that intervention for abnormal bleeding must be focused on improving the QOL and not just controlling the blood loss.^3^ Thus, instruments have been suggested for both aspects (blood loss and QOL).

The dosage of alkaline hematin (AH) and graphical methods are widely used for quantitative evaluation. These tools correlate the visual appearance of the loss of menstrual fluid in standardized sanitary products to the volume estimated in milliliters.^10^ Examples are the Pictorial Blood Assessment Chart (PBAC) and menstrual pictogram.^11–13^ When menstrual symptoms are only assessed, there is a questionnaire called The Menstrual Evaluation Questionnaire, which includes statements about menstrual symptoms.^14^

However, these tools have limitations. AH requires the storage of sanitary products for further laboratory analysis; graphic methods do not include sanitary products, such as menstrual collectors, diapers, cloth pads, and menstrual panties. Moreover, instruments that exclusively assess symptoms cannot objectively assess the blood loss. Additionally, such instruments do not assess the possible social impact, may be affected by the patient's memory, do not distinguish between chronic and intermittent symptoms, or specifically assess the QOL.^10^

There are general questionnaires to assess the QOL, such as the Medical Outcomes Study 36- Item Short Form Heath Survey (SF-36) and the World Health Organization Quality of Life – abbreviated version (WHOQOL-bref); questionnaires for AUB-specific causes, such as uterine fibroids, have been published.^14,15^ Specific questionnaires capable of assessing menstrual symptoms and the impact of AUB on women's QOL have been studied, such as the Menorrhagia Multiattribute Scale, the Menstrual Impact Questionnaire, and the Menstrual Bleeding Questionnaire (MBQ); none of them are validated for use in Brazil.^16–19^ Comparative analyzes between these instruments suggest that they are capable of quantifying the blood loss and providing a qualitative assessment of the QOL.^10^

## OBJECTIVE

This study aimed to translate and culturally validate the first instrument capable of associating both qualitative and quantitative assessments of AUB for use in Brazilian women, in addition to assessing a cut-off point for the diagnosis of AUB.

## METHODS

### Translation and validation of the MBQ

We have followed the methodology described in the Guidelines for the process of intercultural adaptation of self-report measures^20^ and the Guidelines for Reliability and Agreement Study Reports.^21^ Permission and consent for translation and validation of the MBQ was obtained by email from Dr. Matteson.^19^ Subsequently, the MBQ was translated from English into Brazilian Portuguese, through notarized translation, by two different native translators from Brazil with proficiency in English and official authorization to translate scientific documents One translator knew the questionnaire concepts, whereas the second translator did not. Subsequently, the synthesis of the two translated versions was performed, which was back-translated to English (“back-translation”) by a third translator, who was unaware of the original version of the MBQ. After confirming agreement between the retranslated instrument and its original version, the MBQ was analyzed by an expert panel (GPR, LGOB, and CLBP). A face-to-face pre-test was then carried out (preliminary pilot testing) with the application of the MBQ in 30 women to assess the need for new adaptations (which were not necessary). After completing all recommended steps, the instrument was applied for validation.

### Study design and participants (inclusion/exclusion criteria)

A prospective cohort study was carried out at a tertiary, academic-affiliated, outpatient clinic at the Department of Obstetrics and Gynecology, School of Medical Sciences, University of Campinas, Brazil, including women diagnosed with AUB (case group) and women without the criteria for AUB (controls), who were native Brazilians and fluent in Brazilian Portuguese, invited to participate voluntarily, coming from urban or rural areas, and who had already scheduled appointments. Women who agreed to participate signed informed consent forms. This study was approved by the Institutional Review Board (CAAE number 24742619.4.0000.5404).

The inclusion criteria comprised women with AUB (case group; n = 100) presenting complaints of increased bleeding, considering frequency, duration, regularity, flow volume, and intermenstrual bleeding, according to the 2018 International Federation of Gynecology and Obstetrics (Federação Internacional de Ginecologia e Obstetrícia [FIGO]) for at least 6 months (Table 1).^6^ For the control group (n = 100), women without any history of menstrual disorders since menarche and without complaints of AUB were included, that is, with menstrual cycle within the limits considered normal according to the same criteria of FIGO (frequency of cycles between 24 and 38 days, duration of flow less than or equal to 8 days, adequate blood volume, according to the woman's impression, and absence of intermenstrual bleeding). The controls were not matched to the patients. For both groups, the menstrual pattern reported by participants was considered before using any contraceptive method, excluding iatrogenic causes of AUB or normal cycles secondary to anovulatory methods, such as combined oral contraceptives. In both groups, women should refer to the use of only regularly sized pads to reduce bias, and be between 18 and 55 years of age, following criteria used in the elaboration of the MBQ by Matteson.^19^ Patients in the AUB group were recruited from the Abnormal Uterine Bleeding and surgical gynecology outpatient clinics, while control group patients were recruited from the Family Planning outpatient clinic. The exclusion criteria for both groups were as follows: conditions that prevented the reading and/or understanding of the instruments and women diagnosed with AUB already undergoing clinical or surgical treatment (oral contraceptives, intrauterine device of levonorgestrel, laparotomy, laparoscopy, hysteroscopy, or other) in order to avoid treatment bias.

**Table 1:** Clinical characteristics of women with (n = 100) and without (n = 100) abnormal uterine bleeding

	With AUB (mean ± SD) or n (%)	Without AUB (mean ± SD) or n (%)	P
Age (years)	38.45 ± 9.68	30.61 ± 8.49	< 0.001
Weight (kg)	75.29 ± 16.95	67.29 ± 12.50	< 0.001
Height (cm)	163.20 ± 6.39	164.01 ± 5.51	0.413
BMI (kg/m^2^)	28.34 ± 6.39	25.08 ± 5.06	< 0.001
Number of pregnancies	1.43 ± 1.49	0.88 ± 1.39	0.003
Number of abortions	0.15 ± 0.44	0.08 ± 0.27	0.319
Menstrual cycle duration (days)	22.82 ± 6.56	27.81 ± 2.79	< 0.001
Menstrual flow duration (days)	9.70 ± 6.98	4.73 ± 0.89	< 0.001
Sanitary pads used during the menstrual cycle (number)	40.02 ± 44.01	10.09 ± 4.39	< 0.001
Number of months/year when there was a need to change underwear due to blood overflow (0–12)	10.95 ± 3.11	0.93 ± 3.12	< 0.001
Number of months/year when there as a need to change the usual clothes due to blood overflow (0–12)	10.12 ± 3.79	0.48 ± 2.36	< 0.001
Number of months/year when there was a need of changing sheets and bedding due to blood overflow (0–12)	8.21 ± 4.90	0.24 ± 1.70	< 0.001
Quality of life impact (VAS 0–10)	9.11 ± 1.48	3.62 ± 2.88	< 0.001
Bleeding after sexual intercourse	36.00 (36%)	1.00 (1%)	< 0.001
Intermenstrual bleeding	78.00 (78%)	1.00 (1%)	< 0.001
Comorbidities	39.00 (39%)	12.00 (12%)	< 0.001
Anemia	51.00 (51%)	6.00 (6%)	< 0.001
Blood transfusion	2.00 (2%)	0.00 (0%)	0.498

AUB = abnormal uterine bleeding; SD = standard deviation; BMI = body mass index; VAS = visual analogue scale.

### Instruments

#### MBQ

The MBQ^19^ consists of 20 questions, with the evaluation of four main domains - quantity (“heaviness”), pain, irregularity, and QOL, providing a score. The higher the score, the more negative the impact of bleeding on the QOL. However, there is no established cutoff for the diagnosis of AUB, with only an average score that allows the characterization of the existence of increased bleeding associated with menstrual irregularities. We aimed to translate and culturally validate the MBQ instrument for Brazilian Portuguese using psychometric variables. We also compared the MBQ with a graphic method (PBAC), to determine whether both tools were correlated.^20,21^

#### PBAC

The visual system represents a graduated series of sanitary pads (external and internal) with drawings representing the amount of menstrual blood. Women were asked to choose the number of pads used in the bleeding cycle according to the amount of blood depicted in the graphic representation; the greater the amount of blood represented in the pad, the higher the score. Traditional graph described by Higham et al.,^11^ with a cut-off of > 100 points, was used. PBAC was used to assess the construct validity of the MBQ regarding quantitative pattern of blood loss.

#### WHOQOL-BREF

Developed by the World Health Organization to assess the QOL, and modified as a 26-question tool divided into four domains: physical, psychological, social relationships, and environment. It can be used in healthy populations and in those affected by chronic diseases. The answers followed a Likert scale (1–5); the higher the score, the better the QOL.^23^ It was used to assess the construct validity of MBQ regarding its impact on the QOL.

### Validation - psychometric variables

All women answered a sociodemographic form and questions regarding their menstrual patterns (cycle duration, days of menstrual flow, number of pads used, need to change clothes due to blood overflow during menstruation, occurrence of bleeding after sexual intercourse, and intermenstrual bleeding). Furthermore, information regarding the history of anemia, need for blood transfusion due to uterine bleeding, and self-perceived impact of menstruation on QOL were collected. The MBQ, PBAC, and WHOQOL-BREF instruments were used for all women. MBQ and PBAC were reapplied to 30 women, randomly selected from the sample four weeks after the first interview and without any intervention, to assess test-retest. The MBQ was reapplied to 37 women from the AUB group, four weeks after starting treatment, which could be oral combined contraceptives, oral progestins, levonorgestrel intrauterine device, anti-inflammatory or antifibrinolytic, to assess the responsiveness.^20,21^ Other psychometric variables were internal consistency (degree of interrelationship between items), content validity, “floor” and “ceiling” effect (how much the content of a measure is adequate to reflect global content), discriminant validity between case and control groups, and construct validity (fundamental form of instrument validation, as it checks whether the test measures an attribute or quality that is not operationally defined).

### Statistical analysis

There was no defined sample size pattern for the validation studies. For variables, such as test-retest and responsiveness, the ideal is a minimum of 30–60 cases. Therefore, a sample of 200 participants was used and divided into 100 cases and 100 controls.^24^

Categorical variables are described as absolute and percentage frequency values (n/%), and numerical variables are described as mean and standard deviation values. To compare the categorical and continuous variables between the case and control groups, the chi square or Fisher's exact test and Mann–Whitney's non-parametric test were used.

For internal consistency, Cronbach's alpha and the correlation coefficient were calculated, and in the test-retest, the intraclass correlation coefficient (ICC) was used to assess temporal stability. This coefficient was used to verify the homogeneity (accuracy) of the items. Values above 0.70 indicate adequate internal consistency.^20,21^ The test-retest reliability (reproduction of repeated measures with similar responses by respondents, which assesses temporal stability) was assessed using the ICC. Values ≥ 0.70 signified adequate reliability.^20,21^ In the analysis of internal consistency and test-retest, the MBQ and PBAC scores obtained for both groups at first application and reapplication after 2–4 weeks, without any intervention, were compared, using the Wilcoxon test.

Content validity, “floor” and “ceiling” effect is considered for both effects when at least 15% of the scores are below or above the end of the scale. An instrument with adequate content validity is considered to have no effect.^20,21^ The construct validity was calculated using the Wilcoxon test and Spearman correlation to compare the MBQ with the PBAC or WHOQOL-BREF. The Wilcoxon test was used to assess the responsiveness (to compare pre- and post-treatment scores).

A receiver operating curve (ROC) analysis was used to obtain the cutoff point for MBQ score capable of discriminating the presence of AUB. The significance level was set at 5% (P < 0.05) for all calculations. All data were tabulated in Microsoft Excel using a spreadsheet and analyzed using SAS version 9.4 program (Cary, North Carolina, United States).

## RESULTS

Table 1 presents the baseline characteristics of the women included in the study. Women with AUB presented a higher mean age (38.4 ± 9.6 versus 30.6 ± 8. 4 years; P < 0.001) and body mass index (28.3 ± 6.3 versus 25.0 ± 5.0 kg/m^2^; P < 0.001). They also presented more comorbidities than control group. The most common causes of AUB were leiomyomas (29%) and adenomyoses (15%). Women with AUB presented shorter menstrual cycles (22.8 ± 6.5 versus 27.8 ± 2.79 days; P < 0.001, longer blood flow duration (9.7 ± 6.9 versus 4.7 ± 0.8 days; P < 0.001) and used more menstrual pads per menstrual cycle (40.0 ± 44.0 versus 10.0 ± 4.3; P < 0.001). Additionally, women with AUB more frequently reported the association of more than one type of sanitary product to contain the bleeding, with a higher frequency of changing their underwear (95% versus 9%; P < 0.001), change of usual clothes (92% versus 4%; P < 0.001), and change of sheets and bedding (80% versus 3%; P < 0.001) due to blood overflow. The prevalence of bleeding outside the menstrual period and sinus bleeding was also higher in women with AUB (P < 0.001). Although 95% of women with AUB had already sought medical care to control the bleeding, and the diagnosis of anemia was reported in approximately half of the women with AUB (51%), only two women required an intravenous infusion of iron, and two women received blood transfusion in both groups. Almost all women with AUB reported a worsening of their QOL during menstruation (97% versus 27%; P < 0.001).

The highest MBQ and PBAC scores were obtained in the case group (40.1 ± 7.3 versus 7.2 ± 5.7; P < 0.001; and 654.1 ± 750.0 versus 31.5 ± 64.5; P < 0.001, respectively). For the WHOQOL-BREF, the total score (56.5 ± 12.0 versus 65.4 ± 12.9) and psychological (54.9 ± 13.8 versus 63.7 ± 11.8), social (56.0 ± 20.4 versus 69.5 ± 19.2), environmental (61.2 ± 14.8 versus 69.3 ± 16.0), and self-assessment of QOL expectancy domains (55.8 ± 13.9 versus 68.0 ± 16.4, respectively) were worse within women with AUB (P < 0.001), except for the physical domain (Table 2); these data display the discriminant validity for both instruments to differentiate the case and control groups.

**Table 2:** Comparison of the baseline scores of the menstrual bleeding questionnaire, pictorial blood assessment chart, and WHOQOL-BREF questionnaires between women with abnormal uterine bleeding (n = 100) and without abnormal uterine bleeding (n = 100)

Instruments	AUB	Control	P*
MBQ	40.17 ± 7.33	7.22 ± 5.78	< 0.001
PBAC	654.14 ± 750.04	31.59 ± 64.52	< 0.001
WHOQOL-BREF (total score)	56.50 ± 12.08	65.48 ± 12.95	< 0.001
WHOQOL-BREF (physical)	54.39 ± 11.29	56.75 ± 12.43	0.138
WHOQOL-BREF (psychological)	54.92 ± 13.86	63.79 ± 11.81	< 0.001
WHOQOL-BREF (social)	56.08 ± 20.41	69.50 ± 19.22	< 0.001
WHOQOL-BREF (environment)	61.25 ± 14.86	69.38 ± 16.03	< 0.001
WHOQOL-BREF (self-perception)	55.88 ± 13.93	68.00 ± 16.41	< 0.001

WHOQOL-BREF = World Health Organization Quality of Life, abbreviated version; AUB = abnormal uterine bleeding; MBQ = menstrual bleeding questionnaire; PBAC = pictorial blood assessment chart.

* Mann–Whitney test.

Regarding the psychometric variables for the MBQ, Cronbach's alpha coefficient was significantly above 0.70 for the total sample, by group, and in the case group retest demonstrated internal consistency (Table 3). There was no significant difference between initial application and the reapplication of MBQ and PBAC between women with AUB without intervention and women in the control group, indicating the test-retest reliability of both questionnaires (P = ns). The agreement between the questionnaires was also verified using the ICC (Table 4). Regarding the content validity, none of the women in the case group had a maximum score (75 points) on the MBQ, and only four women in the control group had a minimum score (4%), with no ceiling or floor effects. Construct validity was demonstrated by the correlation between the total MBQ score and clinical characteristics of the menstrual cycle, PBAC; however, for the AUB group, the MBQ did not correlate with the total score and subdomains of the WHOQOL-BREF (Table 5). Responsiveness was demonstrated before and after treatment using MBQ and PBAC scores (Table 6).

**Table 3:** Internal consistency (20 items) of the menstrual bleeding questionnaire

Groups	n	Cronbach α coefficient
Both groups	200	**0.982**
Women without AUB	100	**0.886**
Women with AUB	100	**0.896**
Retest from Women with AUB	37	**0.987**

AUB = abnormal uterine bleeding.

**Table 4:** Test-retest and internal consistency of the menstrual bleeding questionnaire and pictorial blood assessment chart questionnaires

	With AUB (n = 15)	Without AUB (n = 15)
**MBQ**	38.40 ± 10.53	8.20 ± 3.21
**MBQ reapplied**	38.40 ± 10.43	8.27 ± 3.10
**P** *	1.000	1.000
**ICC**	0.998	0.990
**(95% CI ICC)**	(0.995; 0.999)	(0.971; 0.997)
**PBAC**	273.20 ± 106.99	75.40 ± 41.44
**PBAC reapplied**	272.87 ± 106.31	73.87 ± 39.49
**P** *	1.000	0.250
**ICC**	1.000	0.996
**(95% CI ICC)**	(0.988; 0.999)	1.000; 1.000)

AUB = abnormal uterine bleeding; MBQ = menstrual bleeding questionnaire; ICC = intraclass coefficient; 95% CI = 95% confidence interval; PBAC = pictorial blood assessment chart.

* P value refers to the Wilcoxon test for paired samples between baseline and reassessment tests.

**Table 5:** Spearman correlation between the menstrual bleeding questionnaire score and clinical symptoms of bleeding, pictorial blood assessment chart, and WHOQOL-BREF in women with abnormal uterine bleeding (n = 100)

		r	P
**Menstrual cycle duration (days)**		+ 0.406	< 0.001
**Menstrual flow duration (days)**		+ 0.380	< 0.001
**Sanitary pads used during the menstrual cycle (number)**		+ 0.340	< 0.001
**Months of the year when there was a need of change in underwear due to blood overflow (0–12)**		+ 0.162	0.105
**Months of the year when there was a need of change in usual clothes due to blood overflow (0–12)**		+ 0.240	0.015
**Months of the year when there was a need of change of sheets and bedding due to blood overflow (0–12)**		+ 0.265	0.007
**Bleeding impact on the QOL**		+ 0.438	< 0.001
**PBAC**		+ 0.390	0.001
**WHOQOL-BREF total score**		- 0.151	0.132
	Physical	+ 0.191	0.056
	Psychological	- 0.022	0.824
	Social	- 0.162	0.107
	Environment	- 0.131	0.193
	Self-perception	- 0.141	0.160

WHOQOL-BREF = World Health Organization Quality of Life – abbreviated version; PBAC = pictorial blood assessment chart.

**Table 6:** Responsiveness of the menstrual bleeding questionnaire and pictorial blood assessment chart questionnaires for women with abnormal uterine bleeding (n = 37)

	MBQ score		PBAC score	
	Mean ± SD	P value*	Mean ± SD	P value*
Before treatment	40.65 ± 6.31		443.03 ± 234.72	
After treatment	17.76 ± 18.96		119.69 ± 165.46	
Mean difference	-22.89 ± 18.16	< 0.001	-323.30 ± 235.76	< 0.001

MBQ = menstrual bleeding questionnaire; PBAC = pictorial blood assessment chart; SD = standard deviation.

* Wilcoxon test for paired samples between the baseline and reassessment applications.

**Figure 1:**
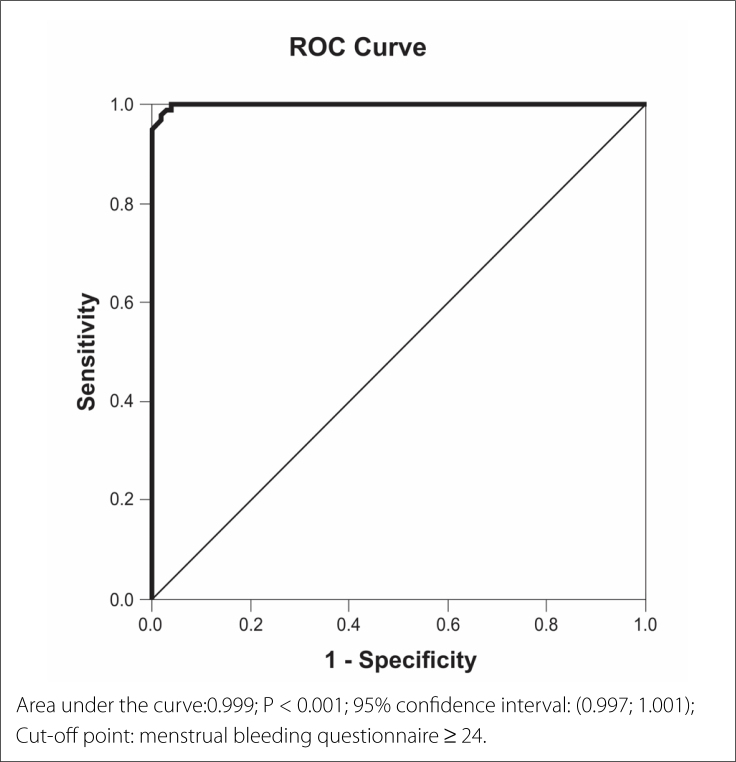
Receiver operating curve using the menstrual bleeding questionnaire score between abnormal uterine bleeding and control women, with a suggested cut-off point to indicate the diagnosis of abnormal uterine bleeding.

To detect an MBQ cutoff point capable of discriminating the presence of increased bleeding, the analysis of the ROC curve indicated that a total MBQ score ≥ 24 was associated with a high probability of abnormal bleeding, with a sensitivity of 98% (95% confidence interval [CI]: 92.26; 99.65), specificity 98% (95% CI: 92.26; 99.65), positive predictive value of 98% (95% CI: 92.26; 99.65), negative predictive value of 98% (95% CI: 92.26; 99.65), and 98% accuracy (95% CI: 94.62; 99.36) (Figure 1).

The final version of MBQ translated and validated can be accessed using the link https://docs.google.com/document/d/170CjydEk0NIIoHpGeWO7IY1qOnIe49Dd8kcN1qLGKJk/edit?usp=sharing.

## DISCUSSION

When treating women with complaints of abnormal uterine bleeding, health professionals must be alert to assess not only the physical repercussions of bleeding, but also the woman's experience with such disorders and its reflection in the worsening of their QOL. The MBQ assesses the AUB quantitatively and qualitatively; however, it has not been validated for use in Brazil. This study showed that the cultural translation of the MBQ is a reliable and valid tool with high internal consistency, temporal stability, construct validity, and responsiveness to treatment. Furthermore, it was easy to use. Our study was also able to calculate a cut-off point for the MBQ instrument, which is capable of accurately discriminating the presence of increased bleeding. The MBQ also allows for the evaluation of response to treatment by comparing the scores before and after the therapy.

The MBQ was validated against one of the most commonly used tools in gynecological practice (PBAC) with high convergent validation. The PBAC is widely used to quantitatively assess the blood loss, owing to its easy understanding, with studies showing that scores between 50 and 185 points are associated with increased bleeding,^22^ with 100 points as the most common cut-off value used in most countries.^11^ Correlation between MBQ scores and those obtained from the PBAC for the total sample and in both groups individually shows that MBQ is an instrument that shows quantitative differences related to uterine bleeding. The original MBQ study proposed scores that discriminated increased bleeding, and whether it was associated with menstrual irregularity. Thus, MBQ scores of 30.8 ± 13.8 were related to increased uterine bleeding, associated or not with menstrual irregularity. The cut-off point suggested by this study (24 points) is compatible with the scores used in the original validation.^19^ Future studies with a larger number of women may reinforce the sensitivity and specificity. Responsiveness of MBQ after AUB treatment strengthened the data. An improvement in the scores after treatment was observed, showing that the MBQ is also a useful instrument for assessing the therapeutic response and follow-up.

However, data from the literature indicate that clinical assessment focused only on quantitative aspects may be insufficient, since the impact of bleeding goes beyond the volume of blood lost, with negative repercussions and impact on the QOL.^25^ Excessive bleeding should be considered when the woman reports the presence of blood loss that interferes with physical, social, emotional aspects, and/or her QOL, emphasizing the importance of qualitative aspects.^22,25^ Thus, considering that the PBAC does not allow evaluation of the impact on QOL or aspects such as the presence of pain during the menstrual period, a generic QOL questionnaire was also utilized (WHOQOL-BREF), due to its rapid application and good psychometric performance.^26^ Specific questionnaires to assess the QOL in women with AUB are scarce, and some are specific to certain causes of AUB, such as the Uterine Fibroid Symptom and Quality of Life (UFS-QOL) for uterine fibroids, and therefore not suitable for use in cases bleeding secondary to other etiologies,^15^ reinforcing the need for a single instrument to assess quantitative and qualitative characteristics of AUB.

In our study, both questionnaires (MBQ and WHOQOL-BREF) showed worse QOL in women with AUB; however, no correlation was found between the total WHOQOL-BREF and MBQ scores in women in the case group. Other studies point in the same direction, such as the original MBQ study, which showed a weak to moderate correlation between the MBQ and the SF-36 and the UFS-QOL validation study, as well as a weak correlation between the two instruments.^15,19^ These findings can be explained by the fact that the MBQ encompasses specific and relevant questions for women with AUB, unlike the SF-36 and WHOQOL-BREF tools, which assess general aspects of the studied population; therefore, they are less specific. Other tools with the approach of all four domains of the MBQ are currently unavailable, and general QOL assessment tools have a global evaluation, which can explain the absence of correlation between these tools. Thus, we believe that the MBQ, as it encompasses situations exclusively related to AUB, may be a preferable tool for assessing QOL during increased bleeding.^10,19^

Considering the high prevalence of AUB in the female population, changes in diagnostic criteria, and the need for tools that help quantitatively and qualitatively in the diagnosis and reassessment of treatment, this study validated the Brazilian Portuguese language as the first questionnaire to assess AUB with a good sample size and compared the MBQ with questionnaires already validated and used to assess AUB and QOL. Another important point is the suggestion of a cut-off point to discriminate AUB and the calculation of the main psychometric variables, demonstrating a robust process of questionnaire validation.^20,21^ As weaknesses: sociodemographic differences between groups may have interfered in the results, is necessary to compare the MBQ with other QOL instruments and short assessment period for responsiveness (four weeks), indicating the need for future studies with women followed up for a longer period with different interventions. The application of the MBQ to a larger population of Brazilian women will also be able to robustly demonstrate our results.

## CONCLUSION

Finally, we believe that the MBQ is a valid, reliable, and stable tool that can be used to assess, diagnose, and follow up AUB treatments in Brazilian Portuguese women. It is important to evaluate women with AUB using validated, standardized questionnaires. Considering the high prevalence of AUB and the economic reality in Brazil, the implementation of a free tool for AUB diagnosis and treatment assessment may help improve the approach to this health condition.
